# Incidence, detection, and tumour stage of breast cancer in a cohort of Italian women with negative screening mammography report recommending early (short-interval) rescreen

**DOI:** 10.1186/1741-7015-8-11

**Published:** 2010-02-01

**Authors:** Alessandra Ravaioli, Flavia Foca, Americo Colamartini, Fabio Falcini, Carlo Naldoni, Alba C Finarelli, Priscilla Sassoli de Bianchi, Lauro Bucchi

**Affiliations:** 1 Romagna Cancer Registry, IRST, 47014 Meldola, Forlì, Italy; 2 Department of Health, Emilia-Romagna Region, 40127 Bologna, Italy

## Abstract

**Background:**

Although poorly described in the literature, the practice of early (short-interval) rescreen after a negative screening mammogram is controversial due to its financial and psychological burden and because it is of no proven benefit.

**Methods:**

The present study targeted an Italian 2-yearly screening programme (Emilia-Romagna Region, 1997-2002). An electronic dataset of 647,876 eligible negative mammography records from 376,257 women aged 50-69 years was record-linked with the regional breast cancer registry. The statistical analysis addressed the following research questions: (1) the prevalence of recommendation for early (<24 months) rescreen (RES) among negative mammography reports; (2) factors associated with the likelihood of a women receiving RES; and (3) whether women receiving RES and women receiving standard negative reports differed in terms of proportional incidence of interval breast cancer, recall rate at the next rescreen, detection rate of breast cancer at the next rescreen and the odds of having late-stage breast cancer during the interscreening interval and at the next rescreen.

**Results:**

RES was used in eight out of 13 screening centres, where it was found in 4171 out of 313,320 negative reports (average rate 1.33%; range 0.05%-4.33%). Reports with RES were more likely for women aged 50-59 years versus older women (odds ratio (OR) 1.33; 95% CI 1.25-1.42), for the first versus subsequent screening rounds (OR 1.91; 95% CI 1.79-2.04) and with a centre-specific recall rate below the average of 6.2% (OR 1.41; 95% CI 1.32-1.50). RES predicted a 3.51-fold (95% CI 0.94-9.29) greater proportional incidence of first-year interval cancers, a 1.90-fold (95% CI 1.62-2.22) greater recall rate at the next screen, a 1.72-fold (95% CI 1.01-2.74) greater detection rate of cancer at the next screen and a non-significantly decreased risk of late disease stage (OR 0.59; 95% CI 0.23-1.53).

**Conclusion:**

The prevalence of RES was in line with the maximum standard level established by the Italian national guidelines. RES identified a subset of women with greater incidence of interval cancers and greater prevalence of cancers detected at the next screen.

## Background

In breast screening, some negative mammography reports recommend that women have their next mammogram at a shorter time interval than the standard one. Radiologists may advise an early rescreen for several, not mutually exclusive, mammographic or patient-reported conditions [1], including radiological abnormalities of doubtful significance [2], the poor technical quality of mammograms [1], previous false-positive results [3], initial versus a subsequent screen (that is, the unavailability of previous mammograms for comparison) [3], high levels of mammographic breast density [1], premenopausal status [1], hormone replacement therapy use [1], a family history of breast cancer [1-4], previous breast cancer [2] including *in situ *carcinoma and a previously diagnosed atypical hyperplasia and benign breast disease [4].

The objective of early rescreen, however, remains the same for all women: to increase the sensitivity of screening for early-stage breast cancer. The earlier the repeat screen, the higher the chance that a new or undetected cancer will be picked-up by a mammogram during its preclinical phase, rather than being diagnosed later as a symptomatic interval cancer or as a late-stage cancer at the standard 2-yearly rescreen.

Although this would seem to be an advantageous option, the practice of early rescreen is controversial. The arguments in favour are that early rescreen would: (1) increase the likelihood of early detection in a subset of women at a high risk of screening failure; (2) reduce the volume of malpractice litigation that results from false-negative mammography findings; (3) limit the recall rate (that is, the proportion of all women undergoing screening mammography who have an abnormal result and are asked to come back immediately for an additional imaging workup) at the initial screen; and (4) avoid excessive increase in the recall rate when trained radiological staff leave the programme because of turnover and are replaced by inexperienced personnel.

Arguments against early rescreen are that: (1) it has been suggested [5] that it is a psychological burden; (2) its benefits for women have not been demonstrated for any condition [4], including radiological abnormalities of doubtful significance; (3) an exhaustive rationale to identify the expected outcome measures of early rescreen has not been developed; (4) an experimental evaluation of effectiveness is difficult to implement within the current public health programmes; (5) the reported or estimated rates of early rescreen vary from as little as 1% in the UK [2] to as much as 10% or more in Canada [3], suggesting that selection criteria are subjectively interpreted; and (6) there is no estimate of whether cost savings related to a decreased recall rate outweigh the extra cost incurred by increased screening frequency.

Given this uncertain background, it is understandable that published practice guidelines and opinions are conflicting. Some experts think that the likelihood of breast cancer for patients with radiological lesions of uncertain clinical significance is high enough to warrant a full diagnostic workup rather than a short-interval repeat mammography [6]. Others, conversely, believe that the criteria for early rescreen predict a risk of disease that is unacceptably low [1]. The European guidelines for breast screening suggest that intermediate mammograms should not be performed [7]. Another view holds that their number should be minimized [4] and that further research is needed in order to assess whether, and which, women with a negative mammogram should be screened more often that every 2 years [3,4]. In Italy, for example, the national guidelines have set an acceptable standard of less than 1% of the screened population [8].

It is quite plausible that inconsistencies and discrepancies among guidelines have generated confusion in the female public and also in the healthcare community. However, this cannot be fully confirmed because the diffusion and practice patterns of early rescreen in the Western countries have been seldom investigated. To the authors' knowledge, only in Australia and Canada are data on early rescreen regularly published as part of statistical reports of screening activities [3,4]. In the USA, the majority of published studies have dealt with a subgroup of women - that is, those with 'probably benign' mammographic abnormalities recommended for short-interval mammography follow-up (American College of Radiology Breast Imaging Reporting and Data System (BI-RADS) category 3) [9-12]. It must be pointed out that the conditions prompting early rescreen in Europe, which may also include high levels of mammographic breast density and non-radiological factors, are not the equivalent of BI-RADS category 3 lesions. However, with the exception of a comprehensive survey of the UK National Health Service screening centres published several years ago [2], no study has ever been published from any European screening programme.

This paper describes a study from a regional screening programme in northern Italy. Our general objectives were to evaluate the prevalence, the epidemiological determinants and the possible outcomes of negative mammography reports containing the recommendation for early rescreen (abbreviated here as RES in partial accordance with Ong *et al*. [2]).

## Methods

### Rationale and research questions

Between 1996 and 1998, as described in detail elsewhere [13,14], a 2-yearly breast screening programme was implemented for women aged 50-69 years living in the Emilia-Romagna Region of northern Italy (*n *= 550,000). We present an additional analysis of mammography records collected for a previous investigation on the proportional incidence of interval breast cancers [14]. The authors of the previous study found a subset of negative results with RES (<24 months) which were pooled with those not otherwise specified.

The rationale and objectives of our study were developed in a three-step process. First, since we found no information on reasons for RES in the above mammography records, we carried out a questionnaire-based interview of the chief radiologists of the district screening units in order to determine which criteria had been used, with whatever frequency, during 1997-2002. In their responses, the following were mentioned: an equivocal mammographic abnormality [2]; a high level of breast density [1]; a strong family history of breast cancer [1-4]; a previously diagnosed atypical hyperplasia [4]; and a previously diagnosed breast cancer [2], including *in situ *carcinoma [4], which referred to breast cancer patients unknown to the screening service and, thus, inadvertently invited.

Second, we established the study rationale. Our approach was that the study endpoints (or the parameters of evaluation of RES) should include the expected outcomes of RES from the radiologists' viewpoint. These were identified based on the following considerations: the conditions identified from interviews are risk factors for breast cancer; breast density [15,16], family history [17] and previous breast cancer [18] are also established risk factors for interval cancer; interval and screen-detected cancers associated with a family history have an increased growth rate [17]; and screen-detected cancers preceded by a mammogram with findings of uncertain clinical significance have a longer stay in the preclinical detectable phase.

Due to these characteristics, women selected by radiologists for early rescreen are expected to have a greater incidence of interval cancer (over an interval of comparable length) and a greater detection rate of cancer on the next screen than women scheduled for a 24-month repeat mammogram. Without early rescreen, these women would also have a greater proportion of late-stage cancers detected either during the interscreening interval or on the next rescreen. Shortening their interscreening interval aims precisely at increasing the likelihood of a new or undetected breast cancer being screen-detected by an early repeat mammogram during its preclinical phase, rather than presenting later as a symptomatic interval cancer or progressing to an advanced stage before 24 months have elapsed. Based on this rationale, we identified the two main endpoints of the study: the prevalence of cancer among women receiving RES; and the proportion of late-stage cancers detected either during the interscreening interval or on the next rescreen.

Third, and closely related to the above, the following research questions were identified: (1) the prevalence of RES among negative mammography reports; (2) factors that are associated with the likelihood of a women receiving RES; (3) whether women receiving RES and women receiving standard negative reports differ in terms of (a) proportional incidence of interval breast cancer in the first and second interval year, (b) recall rate at the next rescreen, (c) detection rate of breast cancer at the next rescreen and (3) odds of having late-stage breast cancer during the interscreening interval and at the next rescreen.

### Research design

The study design was: (1) to follow-up women starting from the point when they had the first mammogram meeting the following requirements - date 1997-2002, woman's age 50-69 years, invitation to screening, negative result with or without RES and unequivocal classification as to the recommended time interval to next screen; (2) to assess the incidence of first- and second-year interval cancers during follow-up; and (3) to use the pool of subsequent mammograms as the denominator for the detection rate. Subsequent mammograms meeting the above requirements were used as a baseline for another interscreening interval and, perhaps, another rescreen.

### Sample

As previously described [14], a dataset containing all the nominative records from women undergoing mammography between 1997 and 2002 was extracted from each of the screening databases of the 13 regional health districts. The datasets were sent to the study coordinating centre in an encrypted format, checked for the conformity of field properties, imported into the Stata software (StataCorp LD, Texas, USA) and merged into a single dataset. This new dataset was composed of 919,538 records from 495,294 women.

### Definitions of interest

Specific methods were used in order to define and identify the negative and positive screening mammography results as well as the interval and screen-detected cancers. In the dataset fields for the indication and result of mammography, the attribute codes were decoded into text descriptions using conversion tables provided by each district screening unit. The final text descriptions were grouped into broad types of indication (screening, diagnostic assessment of a positive screening result, clinical indication) and result (standard negative, negative with RES, positive, unsatisfactory). For the following three subsets of mammograms, the indication and/or the result were defined using *a priori *criteria without consideration of the original classification: (1) even when recorded as screening tests, mammograms performed within 24 months of a positive screening result were assumed to have been performed for diagnostic assessment; (2) by implication, early rescreen mammograms were assumed to have been performed for screening, not diagnostic assessment; and (3) mammograms reported as negative, but with the recommendation for diagnostic assessment, were assumed to be positive.

Among the mammograms for which we retained the original classification of indication and result, a mention should be made of mammograms reported as negative with RES, which we considered a subset of negative results in accordance with Woodman *et al*. [19], and of positive mammograms with negative diagnostic assessment. In the studies of the so-called 'screening episode sensitivity' [20,21] - that is, the combined ability of mammography and of further assessments to identify breast cancers in the screened population, which takes into account the need for diagnostic confirmation after a positive screening result - positive mammograms with negative diagnostic assessment are reclassified as negative. Given our purposes, we did not use this approach and restricted the definition of negative mammogram to those which were originally reported as such.

As far as the detection mode of breast cancer is concerned, we defined an interval breast cancer as an invasive cancer clinically diagnosed within 24 months of an eligible negative mammogram and before the date of the first occurring censoring event (see Data analysis section) [14]. This definition also applied to cancers diagnosed in lapsed attenders (women aged above 69 years with previous eligible negative mammogram) [22] and to cancers occurring in women whose last screening result was negative and who declined subsequent invitations [23].

For the purposes of this study, a screen-detected cancer was defined as an invasive cancer detected within 24 months of a positive rescreen mammogram.

Interval cancers and screen-detected cancers were identified with record linkage of the mammography dataset with the regional breast cancer registry [14]. Interval cancers were identified for the years 1997-2002. In order to identify screen-detected cancers based on a 24-month follow-up of positive results, the mammography dataset was record-linked with the registry files for the years 1997-2004. Record linkage was done using the first name, last name, date of birth, date of the last mammogram and partial combinations of these variables. Manual checks of complete records in the originating databases were carried out for failed matches, partial matches and multiple matches. The breast cancer registry also provided tumour stage information.

### Analysis

#### Prevalence of and factors associated with RES

Descriptive statistics and the χ^2 ^test were used to analyze the prevalence of RES in negative mammography reports (research question No. 1). The independent factors associated with RES (research question No. 2) were identified using a multiple logistic regression model.

#### Incidence of interval cancers

The incidence of interval breast cancers (research question No. 3a) was expressed as a proportion of the underlying incidence or proportional incidence. More exactly, this is the ratio of the number of cancers occurring in the interscreening interval to the number which would be expected if screening had not been offered. For both first and second interval year, women with a negative mammography result were considered at risk of interval cancer until the date of the next screen or the 365th day of interval or 31 December 2002, whichever came first. The number of woman-years at risk was adjusted for the risk of dying - that is, reduced by the expected number of deaths based on the annual age-specific (5-year groups) mortality rates from all causes in the general female population. In order to determine the expected number of cancers, the average age-specific (5-year groups from 50 to 75 years) incidence rates for the years 1991-1995 were applied to the appropriate number of woman-years at risk. The ratio of observed:expected ratios [24] and the 95% confidence interval (CI) were used to compare the proportional incidence observed after RES and that observed after standard negative reports.

#### Recall rate

The recall rate observed at rescreen among women previously receiving RES and that observed among women receiving a standard negative report were compared (research question No. 3b) with the calculation of their ratio with the 95% CI. The ratio was adjusted by the woman's age group.

#### Detection rate of cancer

The detection rate of breast cancer (research question No. 3c) was expressed as the ratio of the number of cancers detected within 2 years of positive mammograms to the expected number based on the underlying incidence or detected:expected ratio. The expected number was calculated by multiplying the average age-specific incidence rates for the years 1991-1995 by the number of women undergoing rescreen. Women previously receiving RES and women receiving a standard negative report were compared with the age-adjusted ratio of detected:expected ratios and the 95% CI [24].

#### Odds of late-stage cancer

A late-stage breast cancer was defined as a lesion >20 mm in size (or pT2 or higher), or with lymph node metastases or distant metastases. Small (pT1) cancers not undergoing axillary surgery were classified as early-stage cancers [25]. A multiple logistic regression model was used to assess the odds ratio (OR) of late-stage versus early-stage disease for breast cancers (interval plus screen-detected cancers) diagnosed among women previously receiving RES versus women scheduled for a standard repeat screen (research question No. 3d).

### Ethics

In accordance with the Italian legislation, no ethical committee approval was sought. The study was an observational one utilizing data obtained from a cancer registry and a public health programme. Approval was obtained from all institutions involved and from the Department of Health of the Emilia-Romagna Region. All personal identifiers were removed from the analysis dataset.

## Results

### Prevalence of and factors associated with RES

The prevalence of RES was 0.64% of the total 647,876 eligible negative mammography reports from the entire study area (Table 1). Restricting analysis to those centres where negative reports with RES were used, the prevalence was 1.33%. RES was more common for younger women, in the first screening round and in those centres with lower recall rate. After inclusion in a multiple logistic regression model (data not shown), these three factors were confirmed to be independently associated with RES. The OR was 1.33 (95% CI 1.25-1.42) for women aged 50-59 years, 1.91 (95% CI 1.79-2.04) for the first screening round and 1.41 (95% CI 1.32-1.50) for a low recall rate. The recommended intervals to early rescreen were distributed as follows: 12 months 73.8%; <12 months 16.6%; and >12 months 9.6%.

**Table 1:** Prevalence of recommendation for early rescreen.

Total screening centres (*n *= 13)	

Women with at least one negative mammography, *n*	376,257
Total negative mammographies, *n*	647,876
Negative mammographies with RES	
*n*	4171
%	0.64

Screening centres using RES (*n *= 8)	

Women with at least one negative mammography, *n*	188,348
Total negative mammographies, *n*	313,320
Negative mammographies with RES	
%	1.33
%, range between centres	0.05-4.33†
%, woman's age 50-59 versus 60-69 years	1.51 versus 1.14‡
%, first versus subsequent screening rounds	2.15 versus 1.08‡
%, centres with recall rate <6.2% versus ≥6.2%*	1.63 versus 1.06‡

*Recall rate was calculated at the screening centre level for the same time period in which the negative mammographies were recorded; 6.2% was the average recall rate in those centres using RES.

† *P *= 0.000 for a χ^2 ^test with seven degrees of freedom.

‡ *P *= 0.000 for a χ^2 ^test with one degree of freedom.

RES, recommendation for early (<24 months) rescreen.

### Incidence of interval cancers

During the first interval year, as shown in Table 2, the proportional incidence of interval breast cancers observed after RES was more than three times greater than that observed after a standard negative result, although the excess risk was of weak significance. During the second interval year (data not shown), the cohort of women with a standard negative result accumulated 198,949 woman-years and generated 172 interval breast cancers. The proportional incidence was 0.36 (95% CI 0.31-0.42). Among women receiving RES (884 woman-years), 2.1 breast cancers were expected but none was observed.

**Table 2:** Proportional incidence of first-year interval breast cancers by type of previous negative mammography report.

	Mammography report	
	
	Standard negative	Negative with RES
Woman-years at risk, n*	272,710	3286
OBS, *n *(rate)†	96 (35.3)	4 (121.7)
EXP, *n*	640.4	7.6
OBS:EXP ratio (95% CI)	0.15 (0.12-0.18)	0.53 (0.14-1.35)
Ratio‡ of the OBS:EXP ratios (95% CI)	1.00 (referent)	3.51 (0.94-9.29)

* Adjusted for general mortality and rounded to the whole-number value.

† Per 100,000 woman-years at risk.

‡ Adjusted for woman's age (5-year groups).

RES, recommendation for early (<24 months) rescreen; OBS:EXP ratio, observed:expected ratio or proportional incidence of interval cancers; CI, confidence interval.

### Recall rate

The number of negative mammography results which were followed by a rescreen mammogram over the available observation time is shown in Table 3. The median time to rescreen was 12 months (25th percentile, or p25, 11 months; p75 14 months) for women receiving RES and 25 months (p25 23 months; p75 28 months) for women with standard negative results. The recall rate for abnormal mammograms was 6.3% and 3.2%, respectively. The ratio between these rates was 1.90 (95% CI 1.62-2.22).

**Table 3:** Detection rate of breast cancer at the next rescreen by type of previous negative mammography report.

	Mammography report	
	
	Standard negative	Negative with RES
Women at rescreen, *n*	127,936	2557
Abnormal results, *n *(%)	4125 (3.2)	161 (6.3)
DET, *n *(rate)*	539 (4.2)	18 (7.0)
EXP, *n*	304.5	5.9
DET:EXP ratio (95% CI)	1.77 (1.62-1.93)	3.04 (1.80-4.81)
Ratio† of the DET:EXP ratios (95% CI)	1.00 (referent)	1.72 (1.01-2.74)

* Per 1000 women.

† Adjusted for woman's age (5-year groups).

RES, recommendation for early (<24 months) rescreen; DET, detected (screen-detected); DET:EXP ratio, ratio of screen-detected cancers to cancers expected based on the underlying incidence; CI, confidence interval.

### Detection rate of cancer

Table 3 also shows the number of screen-detected breast cancers and its ratio to the expected number, which was 1.72-fold greater for women receiving RES.

### Odds of late-stage cancer

The total number of breast cancers detected either during the interscreening interval or at the next rescreen and the proportion of late-stage cancers are shown in Table 4. Women previously given RES had non-significant 40% lower odds of late-stage breast cancer compared with women who had had a standard negative report.

**Table 4:** Odds ratio of late-stage disease for breast cancers detected either during the interscreening interval or at the next rescreen, by type of previous negative mammography report.

	Mammography report	
	
	Standard negative	Negative with RES
Total cancers, *n*	807	22
Late-stage cancers		
*n*	314	5
%	38.9	22.7
Odds ratio* (95% CI)	1.00 (referent)	0.59 (0.23-1.53)

* Adjusted for woman's age (continuous).

RES, recommendation for early (<24 months) rescreen; CI, confidence interval.

## Discussion

### Interpretation of results: prevalence of RES

There are few published data on the prevalence of RES, especially from Europe, with which to compare our results. Our finding of an average regional rate of 0.64% negative reports with RES conforms with the Italian national standard of no more than 1% [8]. In the UK NHS Breast Screening Programme, the observed rate of early rescreen in the same period of our study was 1.1% [2]. In the USA, a rate of about 5% at first screen was documented in large population studies [1,11], which decreased to less than 2% at subsequent screens [11]. In Canada, the estimated rate is above 10% [3].

Factors accounting for RES were not recorded in the screening service databases. According to interviews with radiologists, early rescreen was recommended on the basis of mammographic findings (including abnormalities of doubtful clinical significance and high levels of breast density) and also several patient-reported conditions. There are reasons, however, for assuming that RES was generally due to mammographic findings. First, one of the most common non-radiological factors for RES -that is, a history of breast cancer- was virtually absent from our data as women with this condition were excluded from screening, with the exception of a few who were inadvertently invited.

Second, inherent in the study design was the fact that all interval cancers observed after RES arose within a year of the negative mammography result. There are sufficient data to indicate that early-presenting interval cancers are mainly false-negative cancers for which, by definition, a significant abnormality can be identified on the original screening mammograms [26-28].

Third, as observed elsewhere [3,11], the prevalence of RES decreased from the first to subsequent screening rounds. This suggests that the lack of previous mammograms with which to compare radiological abnormalities of doubtful significance was a major factor for RES. The age pattern of RES added further confirmation. While the prevalence of breast cancer patients and of women with a positive family history increases with age, the prevalence of RES in our data showed the opposite trend, consistent with the age prevalence of women with high levels of breast density (possibly associated with the use of hormone replacement therapy).

Another important factor associated with the prevalence of RES was the recall rate. Early rescreen was more often recommended in screening centres where the recall rate was lower than the regional average. In other words, increasing the proportion of women given RES was, as expected, a sort of compensation for a low recall rate. In a previous work [14], it was suggested that the relatively low incidence of interval breast cancers that is typical for Italian screening programmes [29-31] cannot be properly interpreted without taking into account their high average recall rate. Our data show that the recall rate may also act as an inverse determinant of the prevalence of RES.

### Interpretation of results: occurrence of breast cancer

Negative mammography reports with RES identified a cohort of women who were at greater risk of breast cancer than women scheduled for a standard 24-month repeat mammogram, with a 3.51-fold increase in the proportional incidence of first-year interval cancers (there were no second-year cases because the accumulation of follow-up time virtually ceased after 12 months), and a 1.72-fold increase in the detected prevalence of cancer at the next screen.

Although different, these two numbers indicate risk increases of a similar magnitude. The odds for screen-detected breast cancer increase with the increasing interscreening interval. Based on previously published data [14], the rate of onset of cancer in our cohort of women approximately doubled in the second interval year. As a consequence, a ratio of 1.72 between the detected:expected ratios at 12 months is comparable with a ratio of 3.51 at 24 months. Previous studies [1,9,10,12] of women with BI-RADS category 3 lesions have reported cumulative breast cancer rates of 0.5% [9] to 2% [10], with follow-up periods varying from 6 months [12] to 3 years [9]. In the study by Yasmeen *et al*. [1], the cumulative rate of breast cancer was about twice that in women whose mammograms were described as 'benign' and 'negative'. However, there are several difficulties in comparing their data with ours.

It must be stressed that the true medical rationale of RES is not, or not merely, to select a subset of women with greater prevalence of cancer. As some of the factors leading to RES identify women at increased risk of late-stage cancer, the rationale of short-interval rescreen is to shorten the preclinical detectable phase of new or missed lesions. The ultimate objective of RES is to detect these tumours before they progress to a more advanced stage. In our small sample of cancers, women previously given RES had non-significant 40% lower odds of late-stage breast cancer compared with women who had had a standard negative report. It can be concluded that early rescreen achieved the objective of giving these women at least the same chance of early detection as those receiving standard negative reports. We have not found published data comparable to ours, although there have been papers that have addressed the favourable tumour stage [9] and the good prognosis [6] of cancers detected among women with 'probably benign' abnormalities recommended for short-interval mammography follow-up.

### Limitations in study design

A strength of the study design was the use of multiple outcome measures, in particular the key assumption that the ultimate medical objective of RES is to decrease the odds of late-stage breast cancer both during the interscreening interval and at the next rescreen.

Conversely, the comparison between RES and standard 2-yearly rescreen recommendation was retrospective and non-randomized, which may have introduced a significant bias. The radiological and epidemiological factors leading to RES are indications for more frequent clinical surveillance between screens. Moreover, they may stimulate women's self-surveillance behaviour and increase the perception of the risk and controllability of breast cancer. Altogether, these effects may increase the rate of interval cancers and favour their early, or timely, detection. This is equivalent to saying that, for women with high-risk conditions, there are factors other than early rescreen that could favourably influence tumour stage at diagnosis.

The ideal study design to assess the independent effectiveness of RES would be a prospective, randomized trial in which women diagnosed with (or reporting) those conditions are randomly assigned to different interscreening intervals. However, the feasibility of such a trial is limited by the apparently small number of countries where early rescreen is practised, the low prevalence of eligible women, the expected contamination of all study arms with frequent clinical surveillance and self-surveillance and the likely presence of ethical concerns.

Nevertheless, future studies on this subject, whether based on an experimental or observational retrospective design, should enable us to improve our understanding of some major aspects that warrant further investigation, namely: the accuracy of case definition for women who are candidate for early rescreen; the outcomes of early rescreen as recommended for specific radiological and epidemiological conditions; and the interactive or cumulative effects of multiple exposures to these conditions.

### Limitations in data quality

The present study also had limitations in data quality, the most serious being the lack of recorded information on factors accounting for RES, which was discussed earlier. A second problem was that the electronic records collected did not have a uniform format or coding. However, with an empirical classification of mammographic codes coupled with checks of complete records in the originating databases, we were able to evaluate as many as 647,876 of the 655,175 negative records included in the previous study of interval cancer incidence [14]. Only 7299 (1%) were withdrawn from the present analysis because of unclear information about the interscreening interval recommended.

A third limitation potentially affecting our results lies in the identification of patients with cancer. In Italy, there is no universal identification number by which to establish the identity of individuals and to link records to them. In the present study, breast cancers were registered and classified for detection mode by the local cancer registries in collaboration with the district screening units. After record linkage, the detection mode was checked centrally against the mammography records. Partial and failed matches were returned to the originating centre for further manual inquiries.

Further biases may have arisen from the methods used to estimate the incidence of interval cancers. As far as the observed incidence is concerned, a small number of cancers occurring after a mammogram reported as negative, but followed by diagnostic assessment [32], was excluded from the definition of interval cancer. Also excluded were those cancers observed after a positive mammogram with negative assessment which we considered as interval cancers only for the estimate of the screening episode sensitivity [21]. The expected number of cancers was calculated using the incidence rates observed in the last five pre-screening years, although these rates were probably inflated by opportunistic screening [33]. The method of linear extrapolation of pre-screening incidence time trends to obtain the underlying incidence in the screening years was not used because it tends to amplify this bias [34].

## Conclusions

Based on the above results, we see three reasons for not discouraging the practice of early rescreen in the study area: (1) the prevalence of RES is kept at a level that is sustainable for the screening service; (2) our results on the determinants of RES suggest that decreasing the rate of short-interval mammograms would translate into a further increase in an already high recall rate for diagnostic assessment; and (3), despite potential biases and limitations, our outcome results are at least suggestive of a positive effect of RES on subsequent occurrence and staging of breast cancer. However, as there are still many open questions concerning the independent effects and the optimal practice patterns of early rescreen, we endorse the view that this practice should be minimized [4] until further research establishes its validity [3,4].

## Abbreviations

BI-RADS: Breast Imaging Reporting and Data System; CI: confidence interval; OR: odds ratio; RES: recommendation for early rescreen.

## Competing interests

The authors declare that they have no competing interests.

## Authors' contributions

LB conceived the study. AR, FFo and LB designed the study. AC coordinated the electronic acquisition of data. AR and LB analysed the data. LB wrote the manuscript. FFa, CN, ACF and PSdB contributed to acquisition of data and revised the manuscript. All authors read and approved the final version of the manuscript.

## Pre-publication history

The pre-publication history for this paper can be accessed here:

http://www.biomedcentral.com/1741-7015/8/11/prepub
